# Supplementary material for: Inherited Hypertrabeculation? Genetic and Clinical Insights in Blood Relatives of Genetically Affected Left Ventricular Excessive Trabeculation Patients
Source: Life (Basel). 2025 Jan 22;15(2):150. doi: 10.3390/life15020150 (PMC11856360; doi:10.3390/life15020150)
Supplement: Supplementary file 1 [file life-15-00150-s001.zip › Supplementary Material S1.pdf]

Supplementary Material S1

Index Patiens' data

Supplementary Table S1: Index Patients' cardiac MR parameters.

ID	LV_EDV*i* (ml/m²)	LV_ESV*i* (ml/m²)	LV_SV*i* (ml/m²)	LV_EF (%)	LV_TM*i* (g/m²)	LV_TPM*i* (g/m²)	LV_GCS (%)	LV-GLS (%)
MR 01	102.19	49.68	52.51	51.39	93.55	43.18	-21.23	-28.81
MR 02	93.91	34.00	59.91	63.80	93.96	29.36	-20.51	-30.80
MR 03	71.17	25.17	45.99	64.63	57.72	17.32	-24.35	-28.96
MR 04	72.68	23.77	48.91	67.30	59.04	19.79	-24.45	-26.01
MR 05	75.49	22.13	53.36	70.68	59.17	19.76	-28.15	-32.20
MR 06	94.23	56.79	37.45	39.74	98.36	40.72	-12.85	-15.71
MR 07	53.88	20.48	33.40	61.99	56.78	21.63	-21.74	-27.69
MR 08	72.31	24.00	48.31	66.81	69.54	23.14	-22.29	-25.49
MR 09	69.43	22.77	46.66	67.21	50.47	15.70	-24.25	-26.96
MR 10	65.68	25.36	40.31	61.38	78.39	31.28	-20.19	-27.39
MR 11	62.48	16.98	45.50	72.82	53.56	18.96	-23.71	-37.55
MR 12	92.21	50.97	41.24	44.73	94.27	39.79	-16.38	-15.47
MR 13	88.15	31.15	57.00	64.66	82.28	24.71	-23.55	-29.99
MR 14	52.57	15.85	36.72	69.84	47.32	14.06	-26.06	-27.08
MR 15	108.34	48.33	60.02	55.4	92.87	40.41	-18.62	-20.29
MR 16	73.69	21.16	52.53	71.29	87.22	29.43	-13.96	-27.82
MR 17	66.34	27.86	38.48	58.00	84.34	28.97	-19.54	-31.74
MR 18	144.40	111.96	32.43	22.46	133.31	51.51	-8.24	-8.51

ID: Identification number, MR: index patient magnetic resonance, i: body surface area indexed parameter, LV_EDV: left ventricular end-diastolic volume, LV_ESV: left ventricular end-systolic volume, LV_SV: left ventricular stroke volume, LV_EF: left ventricular ejection fraction, LV_TM: left ventricular total muscle mass, LV_TPM: left ventricular trabeculated and papillary muscle mass, LV_GCS: left ventricular global circumferential strain, LV_GLS: left ventricular global longitudinal strain.

The cardiac MR scans were performed using Philips Achieva and Siemens Magnetom Area 1.5 T MR scanner, the native long- and short-axis recordings were obtained with ECG-gated a balanced steady state free procession (bSSFP) sequence. The slice thickness was 8 mm and 350 mm field of view was used on average. For the evaluation process, the Medis Suite - QMass (version Medis Suite 4.0) analytical software was used on short axis image contours. TM and TPM parameters were determined using the blood - myocardium signal intensity difference based MassK algorithm at 50% threshold setup.

Supplementary Table S2: Indentified pathogenic or likely pathogenic mutations in Index Patients.

ID	Gene symbol	Affected protein	Relation						ACMG class	Varian ID	Transcripct ID	HGVSc	HGVSp
			LVET	HCM	DCM	ACM	ARTM	Cong. CM					
LVET 046	TTN	Titin	+	+	+			+	LP	chr2-179471877-C-A	ENST00000589042	c.53452G>T	p.Glu17818Ter
LVET 040	MYH7	Myosin, Heavy Chain 7	+	+	+	+		+	LP	chr14-23897873-T-C	ENST00000355349	c.1414A>G	p.Ser472Gly
LVET 028	MYBPC3	Myosin Binding Protein C3	+	+	+	+			P	chr11-47353740 G>A	ENST00000545968	c.3697C>T	p.Gln1233Ter
LVET 025	TTN	Titin	+	+	+			+	LP	chr2-179397981-TC-T	ENST00000589042	c.103360del	p.Glu34454-AsnfsTer3
LVET 030	LMNA	Lamin A	*		+	+			LP	chr1-156085004-C-A	ENST00000368300	c.295C>A	p.Arg99Ser
LVET 013	DES	Desmin	*		+	+			P	chr2-220283699-AGC-A	ENST00000373960	c.525_526del	p.Val176Argfs-Ter48
LVET 049	TTN	Titin	+	+	+			+	LP	chr2-179645921-AT-A	ENST00000589042	c.49937G>A	p.Arg16646Gln
LVET 004	TTN	Titin	+	+	+			+	P	chr2-179392275-G-A	ENST00000589042	c.107578C>T	p.Gln35860Ter
	RYR2	Ryanodin receptor 2	*	+		+	+		LP	chr1-237947092-C-T	ENST00000366574	c.12080C>T	p.Thr4027Met
LVET 029	TTN	Titin	+	+	+			+	LP	chr2-179659682-G-C	ENST00000589042	c.1212C>G	p.Tyr404Ter
LVET 023	MYPN	Myopallidin		+	+			+	LP	chr10-69948821-C-T	ENST00000358913	c.2863C>T	p.Arg955Trp
	MYPN	Myopallidin		+	+			+	LP	chr10-69881632-C-G	ENST00000358913	c.437C>G	p.Ser146Cys
LVET 006	TTN	Titin	+	+	+			+	LP	chr2-179427059-AG-A	ENST00000589042	c.83799del	p.Phe27934-SerfsTer3

ID	Gene symbol	Affected protein	Relation						ACMG class	Varian ID	Transcripct ID	HGVSc	HGVSp
			LVET	HCM	DCM	ACM	ARTM	Cong. CM					
LVET 020	TTN	Titin	+	+	+			+	P	chr2-179604901-TG-T	ENST00000589042	c.13058del	p.Pro4353-GlnfsTer14
LVET 045	TTN	Titin	+	+	+			+	P	chr2-179407435-CTTCAA-C	ENSG00000155657	c.97141_-97145del	p.Leu32381-GlufsTer11
LVET 041	SCN5A	Sodium Voltage-Gated Channel Alpha Subunit 5	*		+	+	+		LP	chr3-38597188-G-C	ENST00000413689	c.4501C>G	p.Leu1501Val
LVET 002	TTN	Titin	+	+	+			+	LP	chr2-179536824-C-A	ENST00000589042	c.35101G>T	p.Glu11701Ter
LVET 031	MIB1	MIB E3 Ubiquitin Protein Ligase 1	+		*				LP	chr18-19426998-C-T	ENST00000261537	c.2305C>T	p.Arg769Ter
LVET 016	KCNQ1	Potassium Voltage-Gated Channel Subfamily Q Member 1	*	*			+		P	chr11-2799221-G-A	ENST00000155840	c.1748G>A	p.Arg583His
LVET 014	MYH7	Myosin, Heavy Chain 7	+	+	+	+		+	LP	chr14-23885311-C-T	ENST00000355349	c.4855G>A	p.Glu1619Lys

*ID: Identification number, LVET: Left ventricular excessive tabeculation, HCM: Hypertrophic cardiomyopathy, DCM: dilatative cardiomyopathy, ACM: Arrhythmogenic cardiomyopathy, ARTM: Other arrhytmogenic cardiac diseases, Cong. CM: Frequent congenital cardiomyopathy mutation, +: Gene mutation is associated to the disease, *: not associated but occuring in the literature, ACMG class: Variant classification according to the American College of Medical Genetics (Franklin, Clinvar, Varsome [1-3]), LP: Likely pathogenic, P: pathogenic HGVSc: human genome variation society coding DNA sequence, HGVSp: human genome variation society protein sequence, LVET: left ventricular excessive trabeculation, CMP: other cardiomyopathies*

The genetic background of the primary individuals was determined by a next generation sequencing (NGS) panel (Illumina Truesight Cardio Sequencing Kit, USA, California) containing 174 cardiomyopathy and heart disease genes and compared to human genome assembly number GRCh37. Online ACMG [1] (ClinVar [2], Franklin [3], Varsome [4]) and genetic (OMIM [5,6] and ClinGen [7,8]) databases were used to assess the severity and the relationship to LVET.

1. Richards, S.; Aziz, N.; Bale, S.; Bick, D.; Das, S.; Gastier-Foster, J.; Grody, W.W.; Hegde, M.; Lyon, E.; Spector, E.; et al. Standards and guidelines for the interpretation of sequence variants: a joint consensus recommendation of the American College of Medical Genetics and Genomics and the Association for Molecular Pathology. *Genet Med* **2015**, *17*, 405-424, doi:10.1038/gim.2015.30.

2. Landrum, M.J.; Lee, J.M.; Benson, M.; Brown, G.R.; Chao, C.; Chitipiralla, S.; Gu, B.; Hart, J.; Hoffman, D.; Jang, W.; et al. ClinVar: improving access to variant interpretations and supporting evidence. *Nucleic acids research* **2018**, *46*, D1062-d1067, doi:10.1093/nar/gkx1153. Available online: https://www.ncbi.nlm.nih.gov/clinvar/ (accessed on 2025).

3. Franklin by Genoox. Tel Aviv, Israel. Available online: https://franklin.genoox.com (accessed on 2025).

4. Kopanos, C.; Tsiolkas, V.; Kouris, A.; Chapple, C.E.; Albarca Aguilera, M.; Meyer, R.; Massouras, A. VarSome: the human genomic variant search engine. *Bioinformatics* **2019**, *35*, 1978-1980, doi:10.1093/bioinformatics/bty897. Available online: https://varsome.com/ (accessed on 2025)

5. Amberger, J.S.; Hamosh, A. Searching Online Mendelian Inheritance in Man (OMIM): A Knowledgebase of Human Genes and Genetic Phenotypes. *Curr Protoc Bioinformatics* **2017**, *58*, 1.2.1-1.2.12, doi:10.1002/cpbi.27.

6. Online Mendelian Inheritance in Man, OMIM® by McKusick-Nathans Institute of Genetic Medicine. Johns Hopkins University (Baltimore, MD). Available online: https://omim.org/ (accessed on 2025).

7. The Clinical Genome Resource (ClinGen): Advancing genomic knowledge through global curation. *Genet Med* **2025**, *27*, 101228, doi:10.1016/j.gim.2024.101228. Available online: https://clinicalgenome.org/ (accessed on 2025)

8. Rehm, H.L.; Berg, J.S.; Brooks, L.D.; Bustamante, C.D.; Evans, J.P.; Landrum, M.J.; Ledbetter, D.H.; Maglott, D.R.; Martin, C.L.; Nussbaum, R.L.; et al. ClinGen--the Clinical Genome Resource. *N Engl J Med* **2015**, *372*, 2235-2242, doi:10.1056/NEJMsr1406261.

Supplementary Table S3: Primers used for PCR amplification and Sanger sequencing.

Primer name	Sequence
MYOP c.437C>G F	GAGCCTAACTTCTGCCAGGA
MYOP c.437C>G R	GCAGGGATAGGGATGGGAAC
MYOP c.2863C>T F	TGGTGAGAAACTGGATCTGGA
MYOP c.2863C>T R	TGTCATCTTCCCTACCTTTGGA
TTN c.97141_97145del F	CTGCTTCCTGGTTCTTTGCT
TTN c.97141_97145del R	TCAAAATTGGGTGACTGAACA
SCN5A c.4501C>G F	GTTGGTGCCTTCTCTTTGCA
SCN5A c.4501C>G R	GAGTGTCCTGGGATGCTACA
TTN c.83799del F	GAAAGTGATGGTGGCAGCAA
TTN c.83799del R	CCAGTTCACAGTAGCTTGAGG
TTN c.1212C>G F	GAGACAACGCTGACAACCTC
TTN c.1212C>G R	TTTGGTCTTCAGTTGCTGCT
MIB1 c.2305C>T F	GGGGCAGAGAAGAAGAGTGC
MIB1 c.2305C>T R	CAGGAAAGAAAGGGTCAACACA
MYH7 c.4855G>A F	CCTGTGCCCTGACTGTCTG
MYH7 c.4855G>A R	TGGTGCCTGTATCAAGACACT
TTN c.13058del F	GCCCCTAGTCCCTTCAGAAC
TTN c.13058del R	GAAGGTCCCTTCCCTGTACC
TTN c.35101G>T F	AGTAGTAGAAGCAGAAGTGGAAGA
TTN c.35101G>T R	GGGTTTCTAATCTTCCAAACTGAA
KCNQ1 c.1748G>A F	TCAGAGGTCAGAGGTGGAGA
KCNQ1 c.1748G>A R	CAGGAGCTTCACGTTCACAC
RYR2 c.12080C>T F	GGAACGATTGGCAAACAGAT
RYR2 c.12080C>T R	TTTGTGGAAGTCCCTCTTGG
TTN c.107578C>T F	ATTGATGTTCACTGTGTATCTGC
TTN c.107578C>T R	TACACCATGTTACTTGGCTTGTCTA
LMNA c.295C>A F	CGAGTCTGAAGAGGTGGTCA
LMNA c.295C>A R	TTCCCTCTCTGTCCTGTCTTG
TTN c.3449del F	ACCACAGATCCACTCACAGAT
TTN c.3449del R	GGGCAAAGTATCAGAACCAGT
MYBPC3 c.3697C>T F	CCCAAGATTTCCTGGTTCAA
MYBPC3 c.3697C>T R	TCTGGAAGCTATTGCCCATC
TTN c.53452G>T F	AGATGTCCCTGGTCCAGTTC
TTN c.53452G>T R	GCCACAGCCAAACTTGTTCT
TTN c.103360del F	AGGGACACTTTGCCTGAAGA
TTN c.103360del R	GGAGTTTTCTCTCCTCCTTC
MYH7 c.1414A>G F	CTTCACTGCCTTGACATGGA
MYH7 c.1414A>G R	TGGTGGTTGAAGAACTGCTG

A: Adenine, G: Guanine, T:Thymine, C: Cytosine, F: forward, R: reverse, TTN: Titin, MYH7: Myosin Heavy Chain 7, MYBPC3: Myosin Binding Protein C3, LMNA: Lamin A, DES: Desmin, RYR2: Ryanodin receptor 2, MYPN: Myopallidin, SCN5A: Sodium Voltage-Gated Channel Alpha Subunit 5, MIB1: MIB E3 Ubiquitin Protein Ligase 1, KCNQ1: Potassium Voltage-Gated Channel Subfamily Q Member
