# Supplementary material for: Inherited Hypertrabeculation? Genetic and Clinical Insights in Blood Relatives of Genetically Affected Left Ventricular Excessive Trabeculation Patients
Source: Life (Basel). 2025 Jan 22;15(2):150. doi: 10.3390/life15020150 (PMC11856360; doi:10.3390/life15020150)
Supplement: Supplementary file 1 [file life-15-00150-s001.zip › Supplementary Material S2.pdf]

Supplementary Material S2

Total Family Members' data

Supplementary Table S1: Interobserver agreement for the measured left ventricular functional and strain ultrasound parameters between the two observers.

	Parameter	ICC	CI lower limit	CI Upper limit
**ECHO**	LV_EDV*i* (ml/m^2)	0.92	0.95	0.99
	LV_ESV*i* (ml/m^2)	0.96	0.89	0.99
	LV_SV*i* (ml/m^2)	0.90	0.73	0.97
	LV_EF (%)	0.96	0.92	0.98
	LV_GLS (%)	0.91	0.75	0.97

ECHO: Cardiac ultrasound, ICC: interclass correlation coefficient, CI: 95% confidence interval, LV_EDV: left ventricular end-diastolic volume, LV_ESV: left ventricular end-systolic volume, LV_SV: left ventricular stroke volume, LV_EF: left ventricular ejection fraction, LV_GLS: left ventricular global longitudinal strain, i: body surface area indexed parameter

Supplementary Table S2: Interobserver agreement for the ECG parameters between the two observers.

	Parameter	ICC	CI lower limit	CI Upper limit
**ECG**	Frequency	0.95	0.96	0.98
	P duration (ms)	0.89	0.77	0.94
	P amplitude (mV)	0.79	0.09	0.93
	PQ duration (ms)	0.96	0.92	0.98
	QRS duration (ms)	0.97	0.88	0.99
	SLI_LV (mm)	0.97	0.95	0.99
	SLI_RV (mm)	0.95	0.88	0.98
	CVC (mm)	0.94	0.91	0.94
	QT duration (ms)	0.86	0.73	0.93
	QTc (Bazett) (ms)	0.87	0.75	0.94
	T duration (ms)	0.77	0.54	0.89
	T amplitude (mV)	0.94	0.87	0.97

ECG: Electrocardiogram, ICC: interclass correlation coefficient, CI: 95% confidence interval, SLI_LV: left ventricular Sokolow index, SLI_RV: right ventricular Sokolow index, CVC: Cornell Voltage Criteria index, QTc (Bazett): Bazett's formula corrected QT time

Supplementary Table S3: Baseline ECG and ECHO values and characteristics of the Total Family Members.

ECG

Abnormal Characteristics on Family Member ECG (n = 55)		
T inversion or biphasic T waves	4	**Total Abnormal Characteristics on ECG** (percentage of Family Member ECG)
Atrial fibrillation or atrial flutter	2	
Recurrent ventricular extrasystoles	2	
Complete left bundle branch block	2	
Incomplete left bundle branch block	3	∑ 15 (27,3%)
Complete right bundle branch block	2	

ECG Values

n	Freq. (bpm)	P dur. (ms)	P amp. (mV)	PQ (ms)	QRS (ms)	SLI_LV (mV)
**55**	73.54 ± 13.35	97.52 ± 14.35	0.11 ± 0.03	153.11 ± 22.45	101.02 ± 15.14	18.12 ± 6.98

SLI_RV (mV)	CVC (mV)	QT (ms)	QTc (Bazett) (ms)	T dur. (ms)	T amp. (mV)
4.33 ± 3.26	11.16 ± 5.89	337.89 ± 31.20	415.50 ± 39.45	172.07 ± 22.78	0.27 ± 0.16

ECHO

ECHO Values

n	LV_EDV*i* (*ml/m^2*)	LV_ESV*i* (*ml/m^2*)	LV_SV*i* (*ml/m^2*)	LV_EF (%)	LV_GLS (%)
51	58.30 ± 12.40	25.28 ± 6.60	34.42 ± 7.71	58.29 ± 4.22	-20.04 ± 2.40

n: total study population number, Freq.: frequency (beat per minute), P dur.: P duration, P amp.: P amplitude, SLI_LV: left ventricular Sokolow index, SLI_RV: right ventricular Sokolow index, CVC: Cornell Voltage Criteria index, QTc (Bazett): Bazett's formula corrected QT time, T amp.: T amplitude, T dur.: T duration, T amp.: T amplitude, LV_EDV: left ventricular end-diastolic volume, LV_ESV: left ventricular end-systolic volume LV_SV: left ventricular stroke volume, LV_EF: left ventricular ejection fraction, LV_GLS: left ventricular global longitudinal strain, i: body surface area indexed parameter.

Supplementary Table S4: Age, sex, genetical and anamnestic data of the Total Family Members.

ID	Age (y)	Sex (male = 1)	Genetical involvement	Subjective symptoms				Anamnestic informations					ECG Abn.
				Syncope	Chest pain	Dyspnoe	Palpitation	Documented Arrhythmia	Non-documented Arrhythmia	Cardiac hospitali-zation	Stroke	Ischemic disease	
LVET002A	14	0	*GEN-neg*	-	+	-	-	-	-	-	-	-	-
LVET002B	47	0	**GEN-pos**	-	+	+	+	+	-	-	-	-	-
LVET002C	49	1	*GEN-neg*	+	-	+	-	-	-	+	-	-	+
LVET004A	14	1	**GEN-pos**	-	+	-	+	+	-	-	-	-	+
LVET004B	65	0	**GEN-pos**	-	+	+	+	-	-	-	-	+	+
LVET004C	45	1	*GEN-neg*	-	+	+	-	-	-	-	-	-	-
LVET006A	32	0	**GEN-pos**	+	-	-	-	+	-	+	-	-	-
LVET006B	36	0	*GEN-neg*	-	-	-	-	-	+	+	-	-	-
LVET006C	32	0	**GEN-pos**	-	-	+	-	-	-	-	-	-	-
LVET006D	57	0	**GEN-pos**	-	+	+	-	+	-	+	-	-	-
LVET013A	56	1	*GEN-neg*	+	+	-	+	+	-	-	-	-	-
LVET013B	20	1	*GEN-neg*	-	+	-	-	-	-	-	-	-	-
LVET013C	24	1	*GEN-neg*	-	-	-	+	-	+	-	-	-	+
LVET013D	63	0	*GEN-neg*	-	-	-	+	+	-	-	-	-	-
LVET013E	61	1	*GEN-neg*	-	-	-	-	+	-	-	-	-	+
LVET013F	51	0	*GEN-neg*	-	-	-	-	+	-	-	-	-	-
LVET013G	65	1	*GEN-neg*	+	+	-	-	+	-	-	-	-	-
LVET014A	29	1	*GEN-neg*	-	-	-	+	-	-	-	-	-	+
LVET014B	24	1	*GEN-neg*	-	+	-	-	-	-	+	-	-	-
LVET016A	79	1	*GEN-neg*	-	+	-	-	-	+	+	-	-	+
LVET016B	83	0	*GEN-neg*	-	-	-	-	-	+	+	-	-	-
LVET016C	25	1	**GEN-pos**	-	-	-	-	-	-	-	-	-	-
LVET016D	54	1	*GEN-neg*	-	-	-	+	-	-	-	-	-	+
LVET016E	61	0	**GEN-pos**	-	-	-	-	-	-	-	-	-	-
LVET020A	36	0	*GEN-neg*	-	-	+	-	-	-	-	-	-	-
LVET020B	34	0	*GEN-neg*	-	+	-	+	+	-	-	-	-	-
LVET023A	23	0	**GEN-pos**	-	-	-	+	-	-	-	-	-	-
LVET023B	76	1	**GEN-pos**	-	-	-	-	-	+	-	-	-	+
LVET025A	46	1	**GEN-pos**	-	-	-	+	-	+	-	-	-	-
LVET025B	53	0	*GEN-neg*	-	-	-	+	+	-	-	-	-	-

LVET025C	23	1	*GEN-neg*	-	-	-	-	-	-	-	-	-	-
LVET025D	82	0	**GEN-pos**	-	+	-	-	-	-	-	-	-	-
LVET025E	89	1	*GEN-neg*	-	-	+	-	-	-	-	-	-	+
LVET028A	42	0	*GEN-neg*	+	+	-	-	-	-	+	-	-	-
LVET028B	13	1	**GEN-pos**	+	-	+	-	-	-	-	-	-	-
LVET028C	26	0	**GEN-pos**	-	+	-	-	-	-	-	-	-	-
LVET029A	32	1	*GEN-neg*	-	-	+	-	+	-	-	-	-	-
LVET029B	73	0	*GEN-neg*	-	-	+	-	-	-	-	-	-	-
LVET030A	41	1	**GEN-pos**	+	+	+	-	-	-	-	-	-	-
LVET031A	45	1	*GEN-neg*	-	+	-	-	-	+	-	-	-	-
LVET031B	68	1	**GEN-pos**	-	-	-	-	-	-	-	-	-	+
LVET031C	7	0	*GEN-neg*	-	-	-	-	-	-	-	-	-	-
LVET031D	67	0	*GEN-neg*	+	-	+	-	+	-	-	-	-	+
LVET031E	18	0	*GEN-neg*	-	+	-	+	-	+	+	-	-	-
LVET040A	41	1	**GEN-pos**	-	-	-	+	+	-	-	-	-	-
LVET040B	38	1	*GEN-neg*	-	-	-	-	-	-	-	-	-	-
LVET040C	8	0	*GEN-neg*	-	-	-	-	-	-	-	-	-	-
LVET041A	70	0	**GEN-pos**	-	-	-	-	-	-	-	-	-	+
LVET045A	38	0	**GEN-pos**	-	-	-	+	-	+	-	-	-	+
LVET046A	31	1	*GEN-neg*	-	-	-	+	-	-	-	-	-	-
LVET046B	29	1	**GEN-pos**	-	-	-	-	+	-	-	-	-	-
LVET049A	46	1	*GEN-neg*	-	-	+	-	-	-	-	-	-	-
LVET049B	38	0	**GEN-pos**	-	-	+	-	-	-	-	-	-	+
LVET049C	13	0	*GEN-neg*	-	-	-	+	-	-	+	-	-	-
LVET049D	42	0	*GEN-neg*	-	-	+	+	-	-	-	-	-	-
**Sum**	**43**	**27**	**21**	**8**	**18**	**16**	**18**	**15**	**9**	**10**	**0**	**1**	**15**

ID: identification number, LVET number and letter: identifies the families and the included family members, y: years, GEN-pos: carries the index patient's mutation, GEN-neg: index patient's mutation could not be identified, ECG abn.: ECG abnormality, Sum: summarised incidence (average in column AGE)

Supplementary Figure S1 (A-M): The genetic pedigree of families.

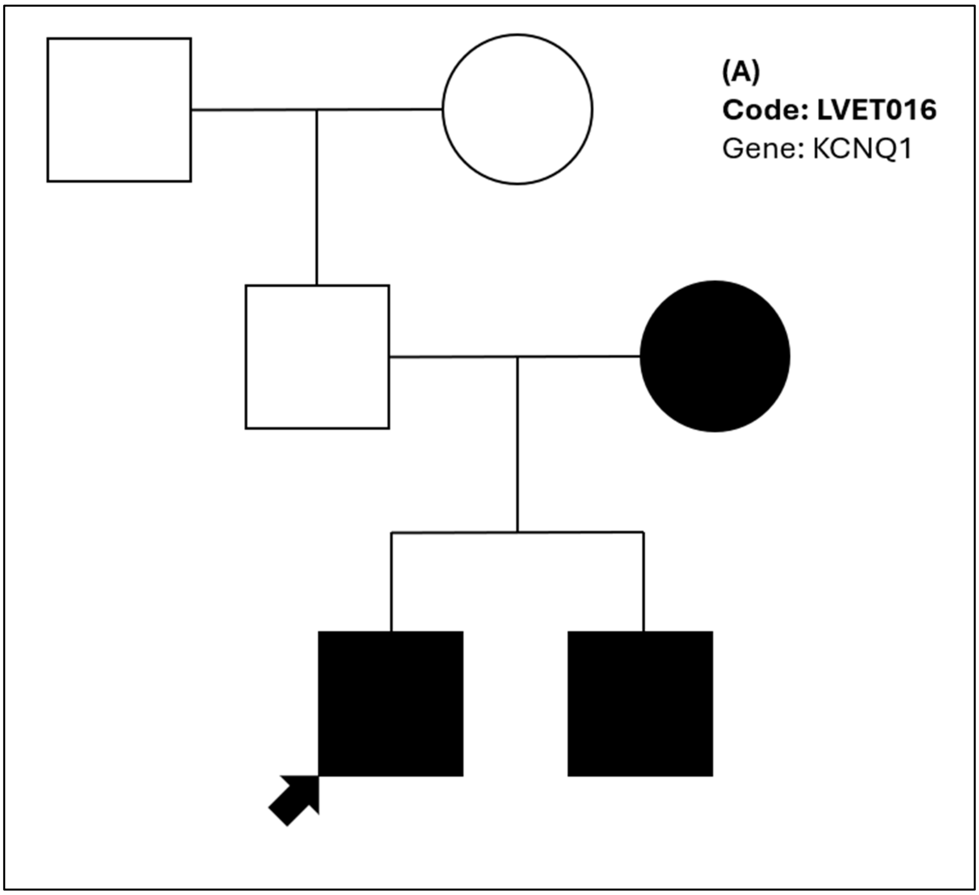

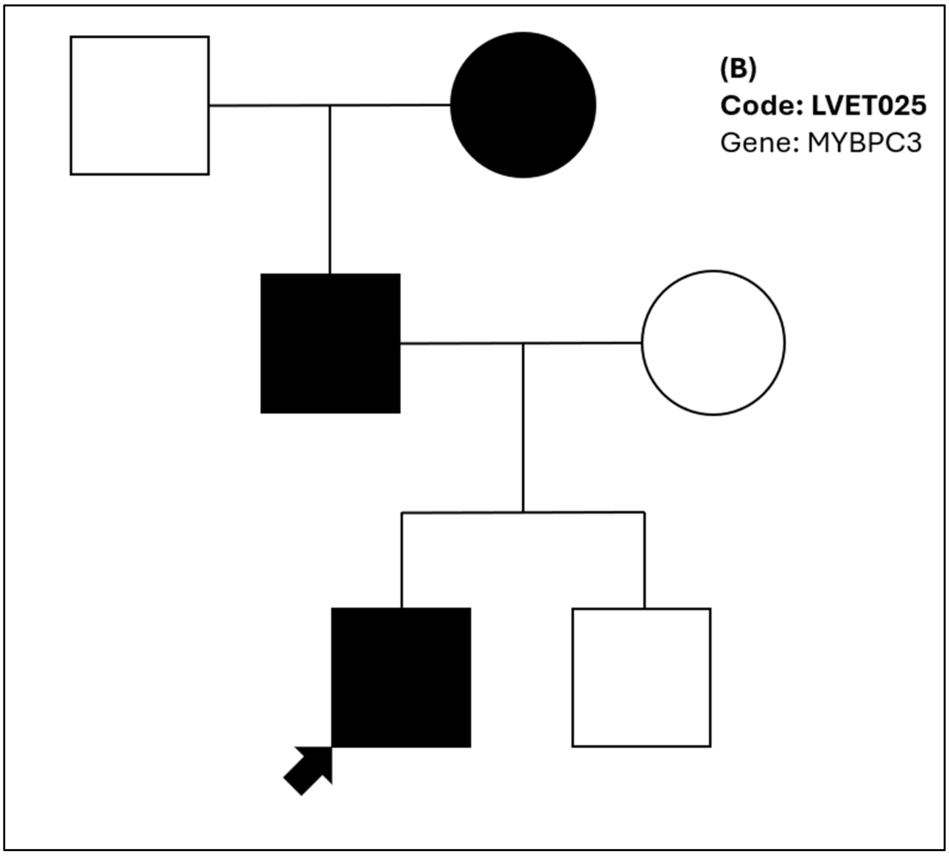

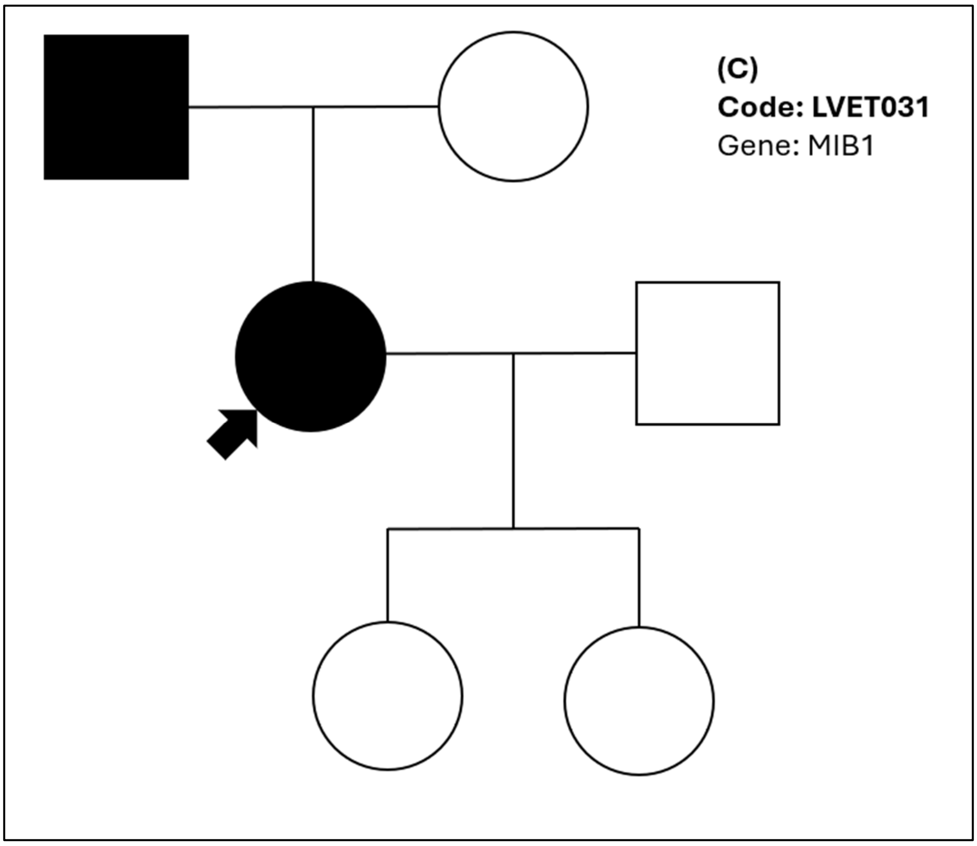
(C)
Code: LVET031
Gene: MIB1

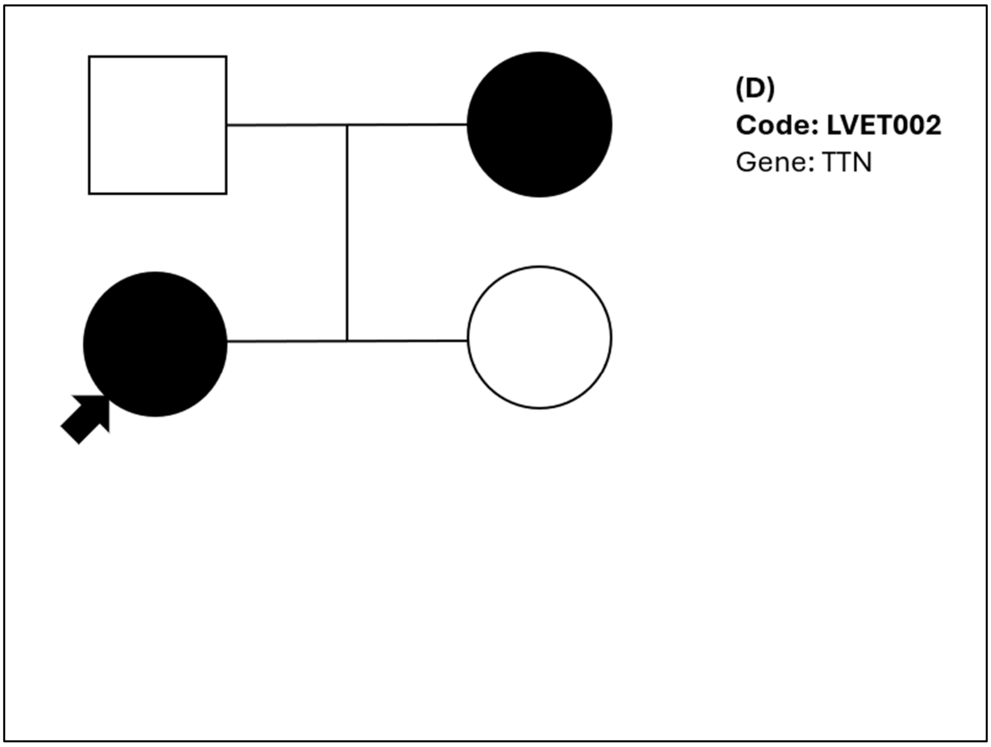
(D)
Code: LVET002
Gene: TTN

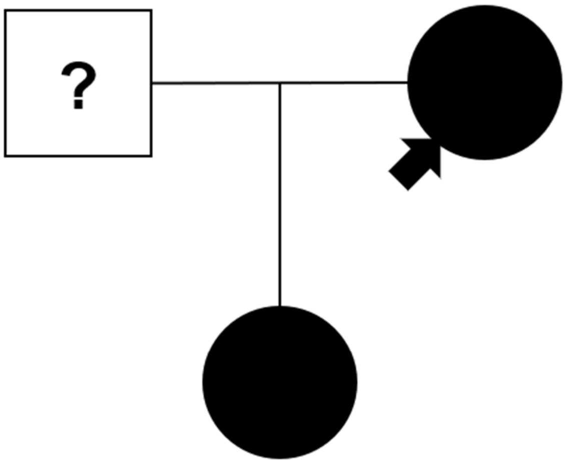

(E)
Code: LVET041
Gene: SCN5A

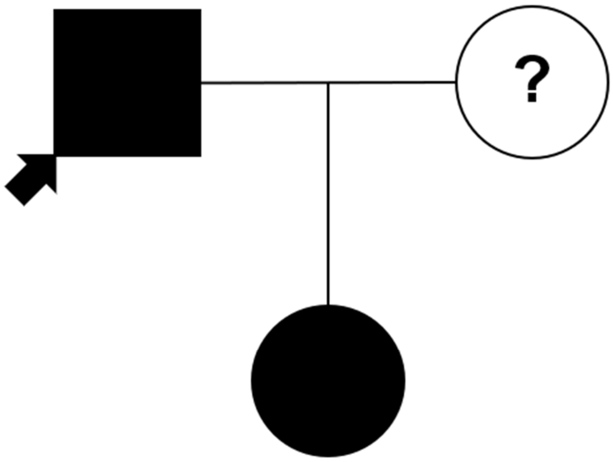

(F)
Code: LVET045
Gene: TTN

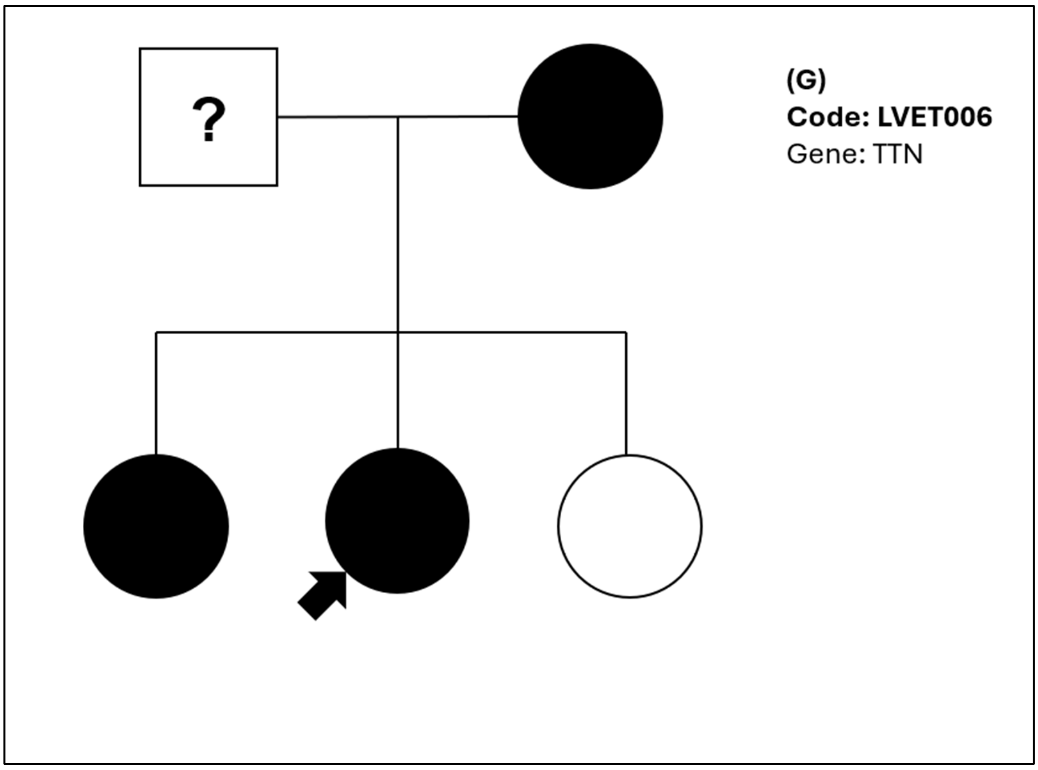
?
(G)
Code: LVET006
Gene: TTN

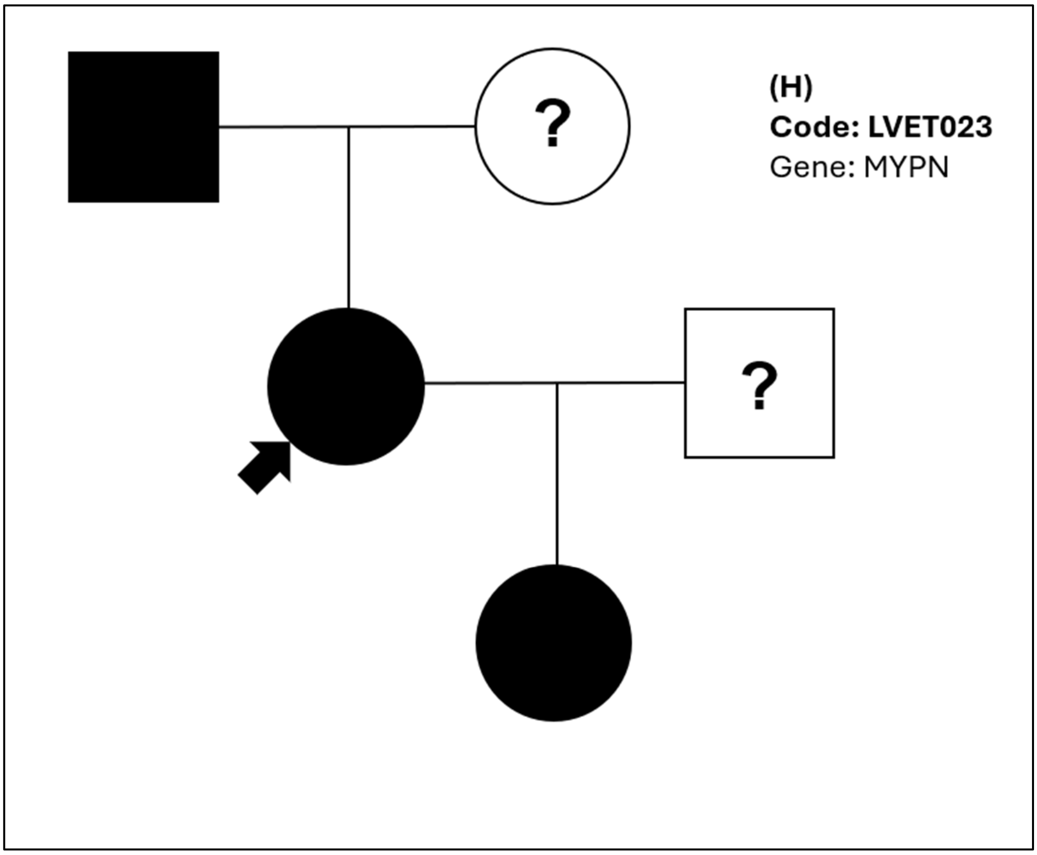
?
(H)
Code: LVET023
Gene: MYPN
?

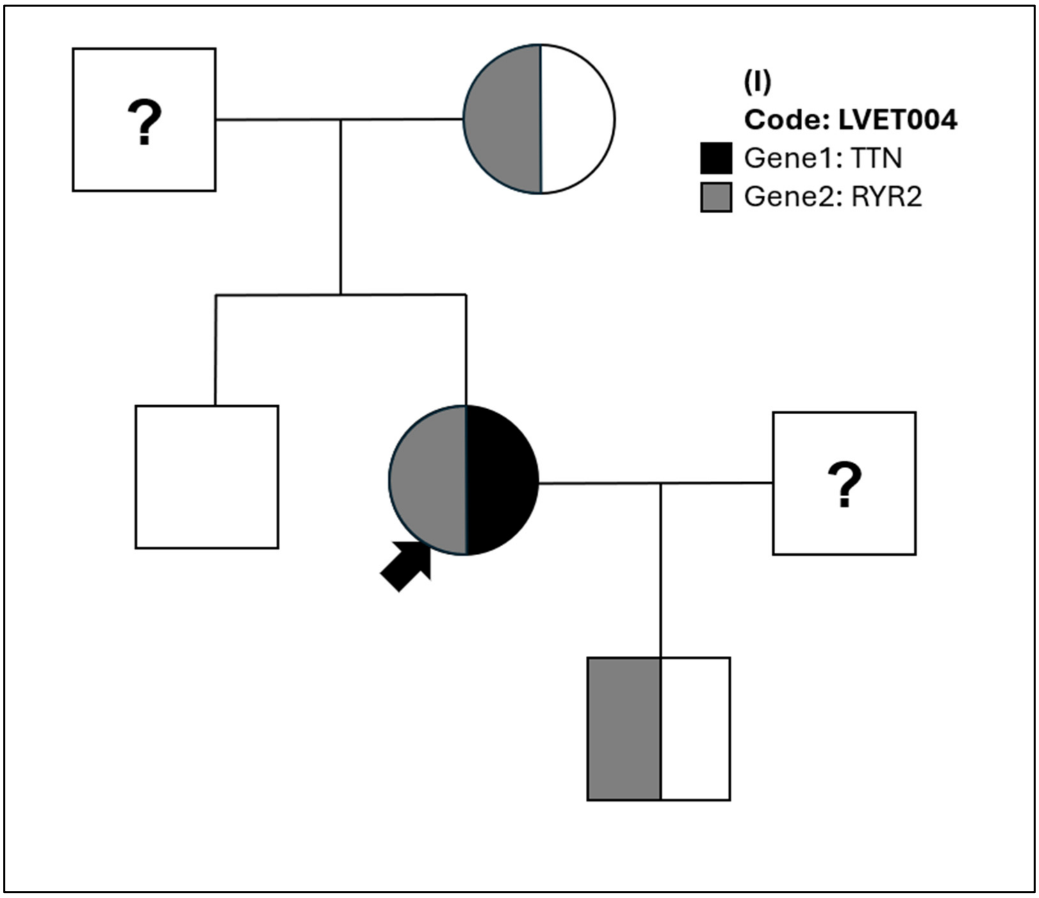
?
?
(I)
Code: LVET004
Gene1: TTN
Gene2: RYR2

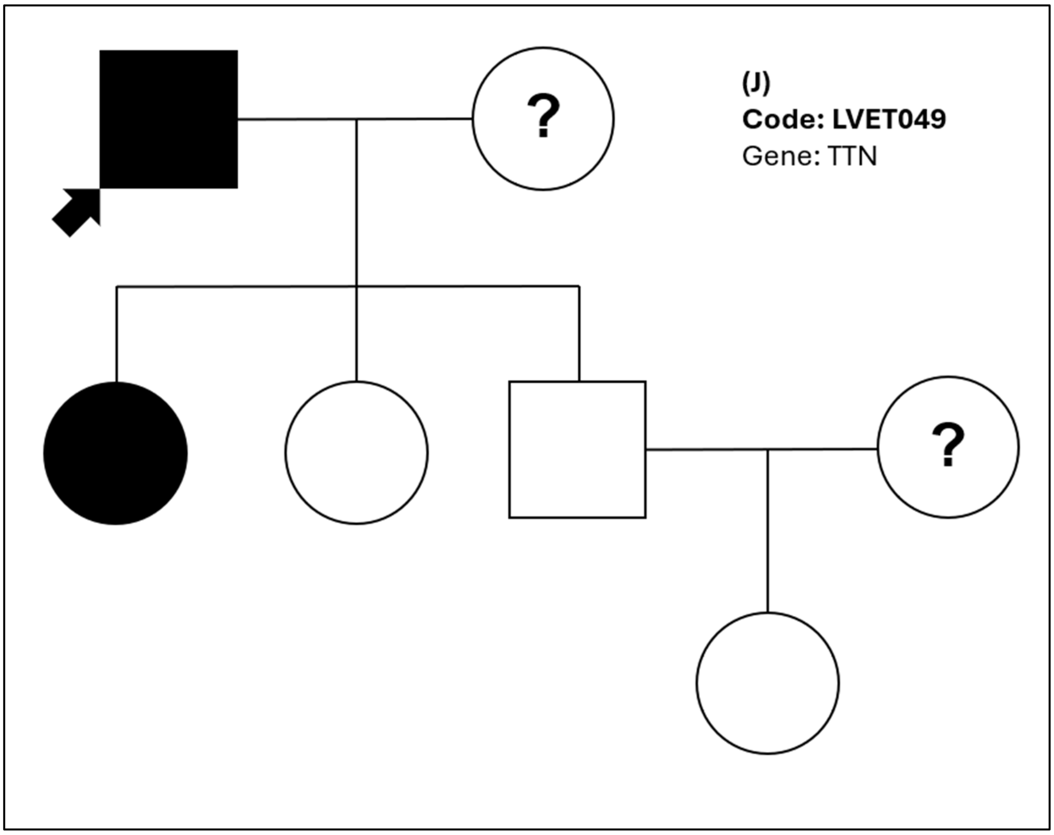
?
?
(J)
Code: LVET049
Gene: TTN

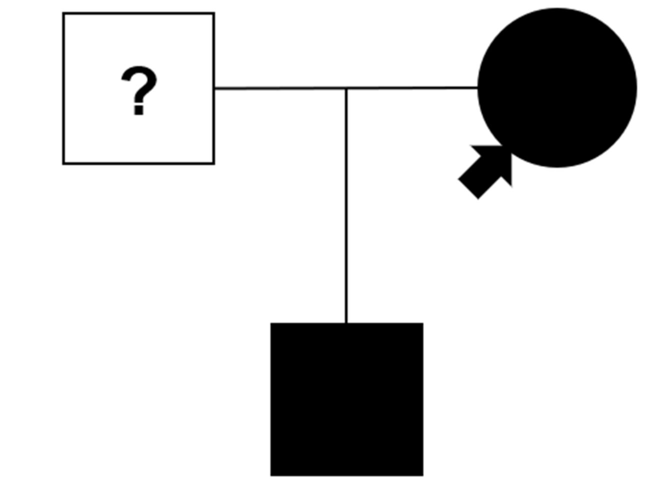

(K)
Code: LVET030
Gene: LMNA

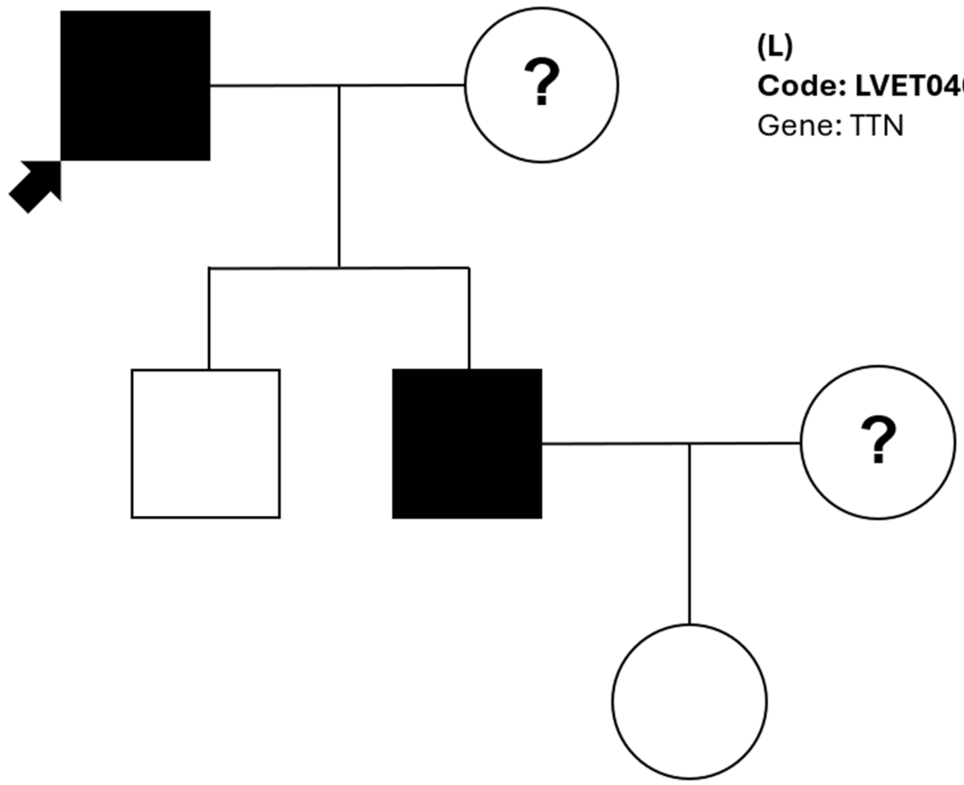

(L)
Code: LVET040
Gene: TTN

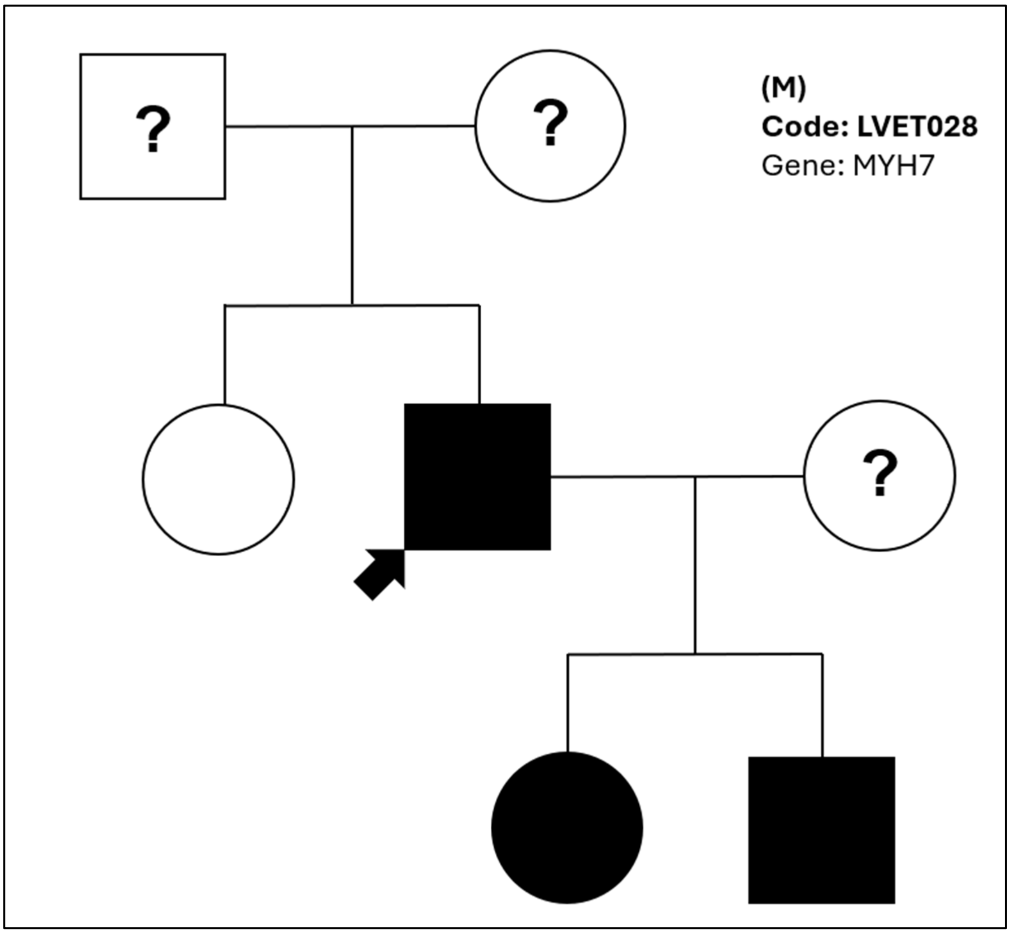

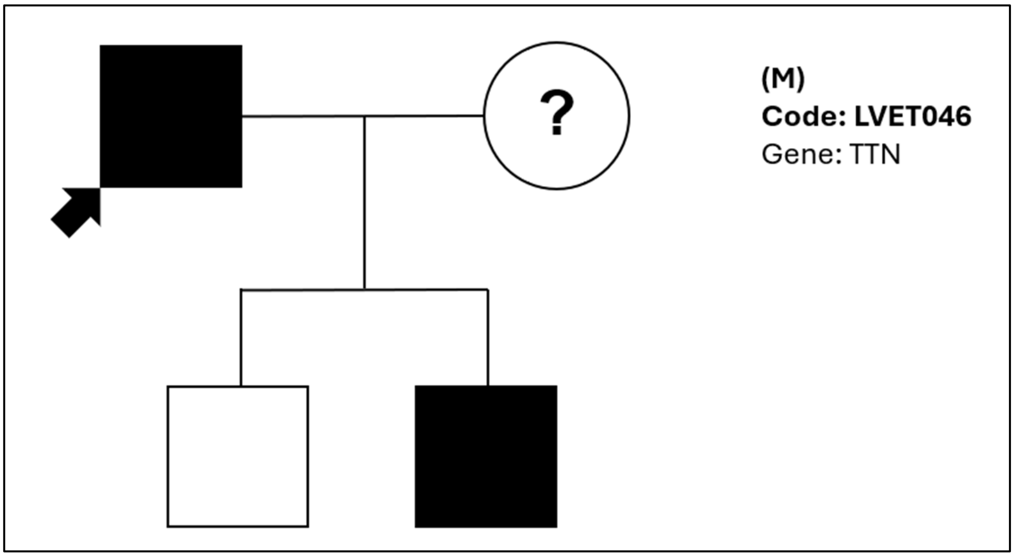

Arrow: Probond (the index patient), ?: unknown genetic background, Circle: female, Square: male, Black/Grey: mutation carrier, White: non-carrier, TTN:Titin, MYH7: Myosin Heavy Chain 7, MYBPC3: Myosin Binding Protein C3, LMNA: Lamin A, DES: Desmin, RYR2: Ryanodin receptor 2, MYPN: Myopallidin, SCN5A: Sodium Voltage-Gated Channel Alpha Subunit 5, MIB1: MIB E3 Ubiquitin Protein Ligase 1, KCNQ1: Potassium Voltage-Gated Channel Subfamily Q Member 1

Supplementary Figure S2 (A and B): Comparison between the genetic subgroups and an age- and sex matched control group in the ECG parameters.

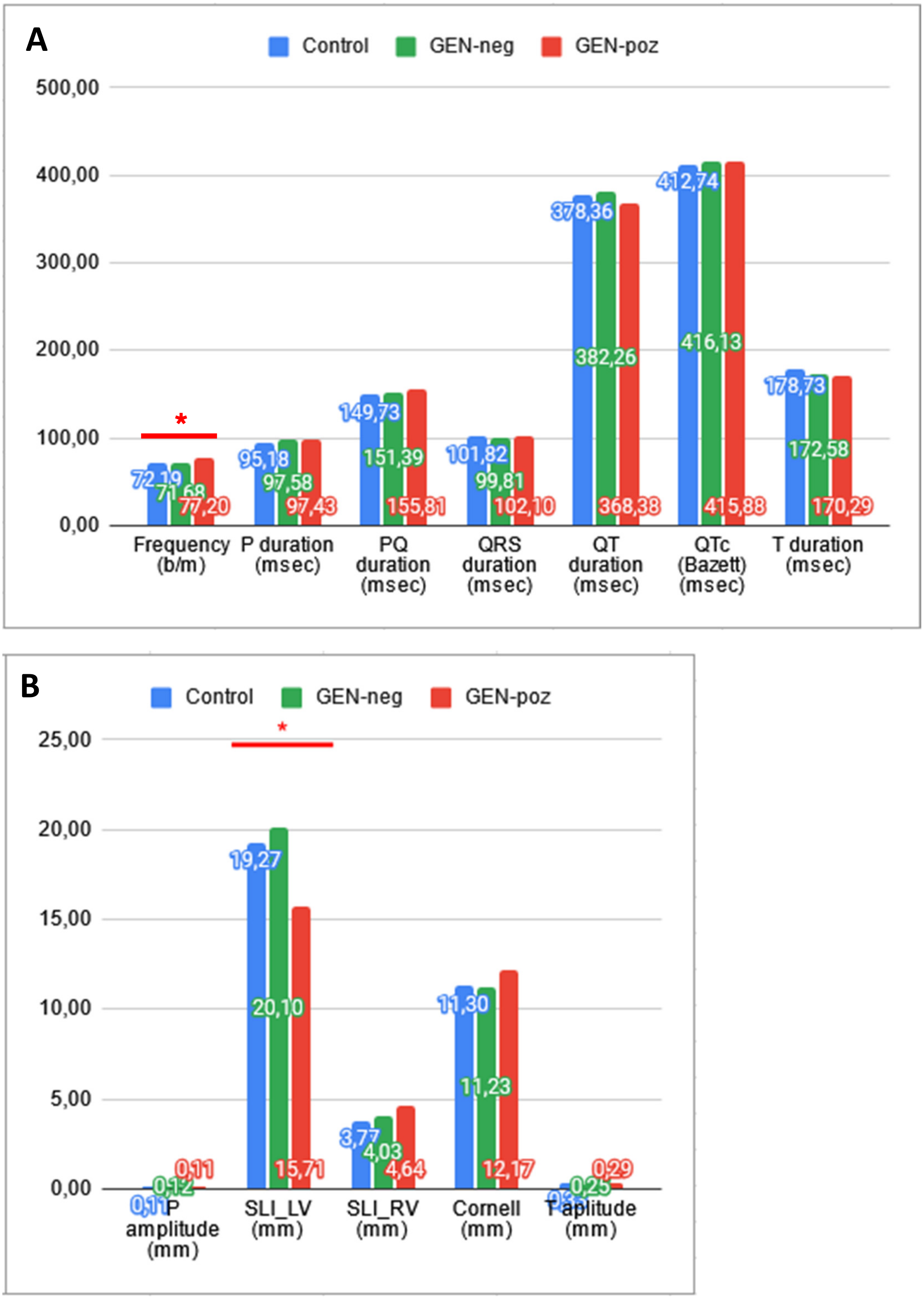

*GEN-neg: family members who do not carry the index patient's mutation, GEN-pos: family members who carries the index patient's mutation, b/m: beat per minute, SLI_LV: left ventricular Sokolow index, SLI_RV: right ventricular Sokolow index, Cornell: Cornell Voltage Criteria index *: p < 0,05 (ANOVA)*

Supplementary Figure S3: Comparison between the genetic subgroups and an age- and sex matched control group in the Cardiac ultrasound (ECHO) parameters.

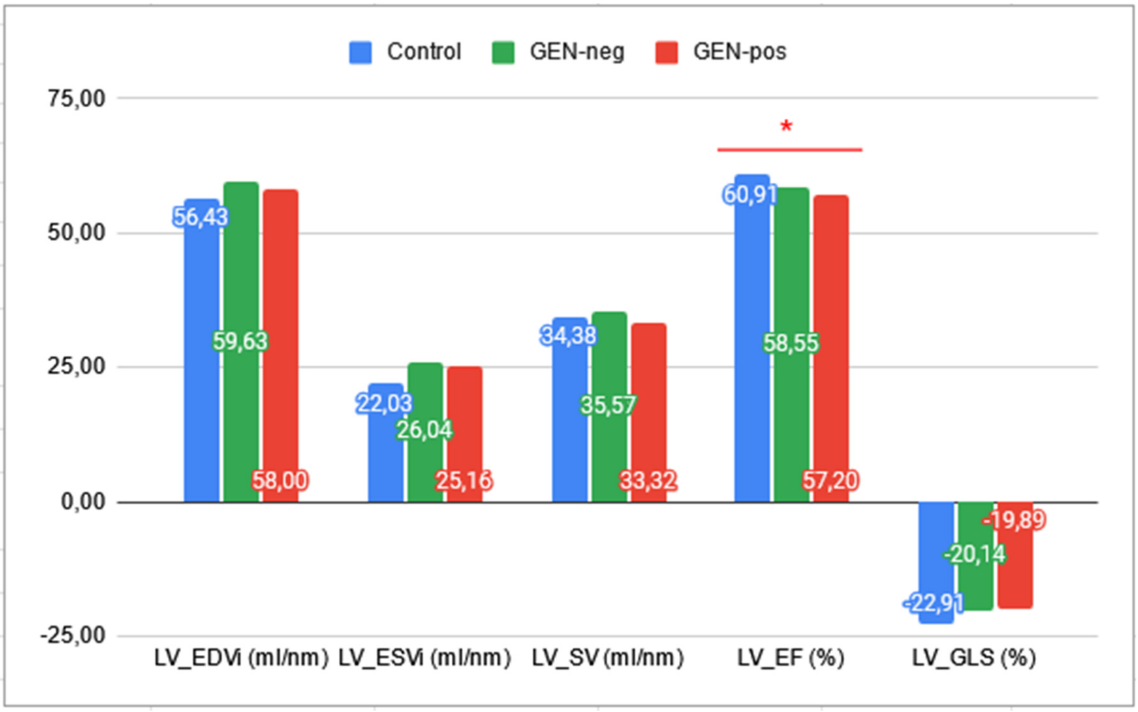

*GEN-neg: family members who do not carry the index patient's mutation, GEN-pos: family members who carries the index patient's mutation, LV_EDV: left ventricular end-diastolic volume, LV_ESV: left ventricular end-systolic volume LV_SV: left ventricular stroke volume, LV_EF: left ventricular ejection fraction, LV_GLS: left ventricular global longitudinal strain, i: body surface area indexed parameter, *: p < 0,05 (ANOVA)*
